# Eclampsia in Brazil in the 21st Century

**DOI:** 10.1055/s-0042-1754378

**Published:** 2022-08-08

**Authors:** José Geraldo Lopes Ramos, Sérgio Hofmeister Martins-Costa, Nelson Sass

**Affiliations:** 1 Department of Gynecology and Obstetrics, Universidade Federal do Rio Grande do Sul, Porto Alegre, RS, Brazil; 2 Department of Obstetrics, Escola Paulista de Medicina, Universidade Federal de São Paulo, São Paulo, SP, Brazil

Recently, Bartal and Sibai 1 wrote about the current concepts of eclampsia in the 21st Century. One of the several interesting points of this article is the comparison of the incidence of eclampsia between developed countries and countries with low socioeconomic status. The eclampsia rate per 10,000 births ranged around 150.6 in Madagascar, 140.1 in Tanzania and 50.2 in India. When comparing these numbers with those of developed countries, we see incredible lower rates such as 8.6 in Australia, 8.4 in Canada, 3.4 in the US, 2.7 in the UK and 1.5 in Finland.

Guida et al. 2 analyzed data on the prevalence of eclampsia in Brazil. The cumulative frequency of hypertensive disease during pregnancy was 6.7%, which is similar to other countries. They found a frequency of eclampsia of 1.7 to 6.2% in hypertensive pregnant women. Of the 10 studies analyzed, 3 reported the occurrence of eclampsia. In these 3 series, we had 42,220 births and an eclampsia rate of 10.42 per 10,000 births. These data point to an eclampsia rate similar to Australia or Canada, but higher than in countries such as the UK and Finland. 1

**Fig. 1:**
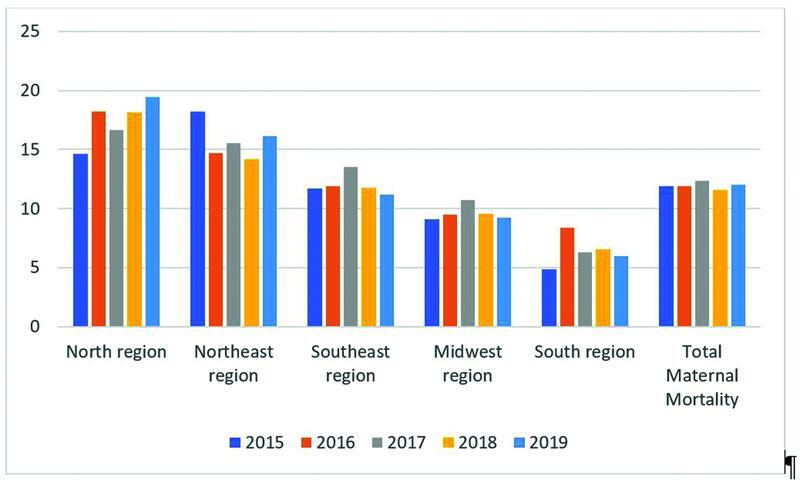
Hypertension mortality rates in Brazil by regions from 2015 to 2019 per 100,000 live births.

**Fig. 2:**
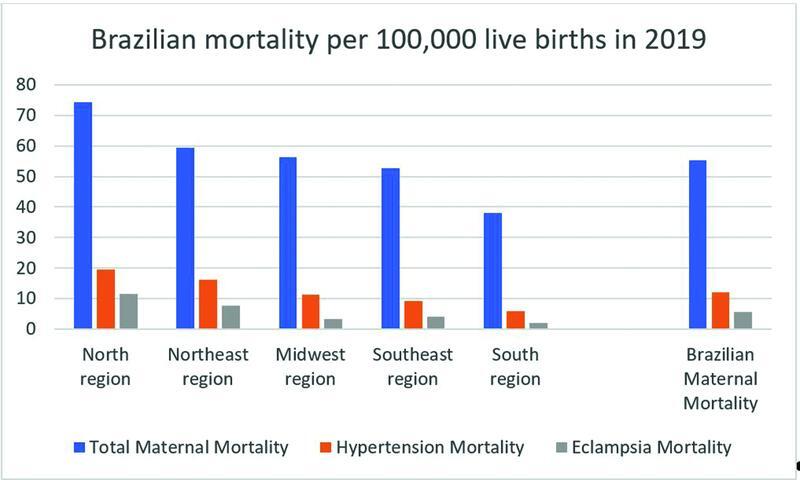
Mortality per 100,000 live births by region in Brazil in 2019.

**Fig. 3:**
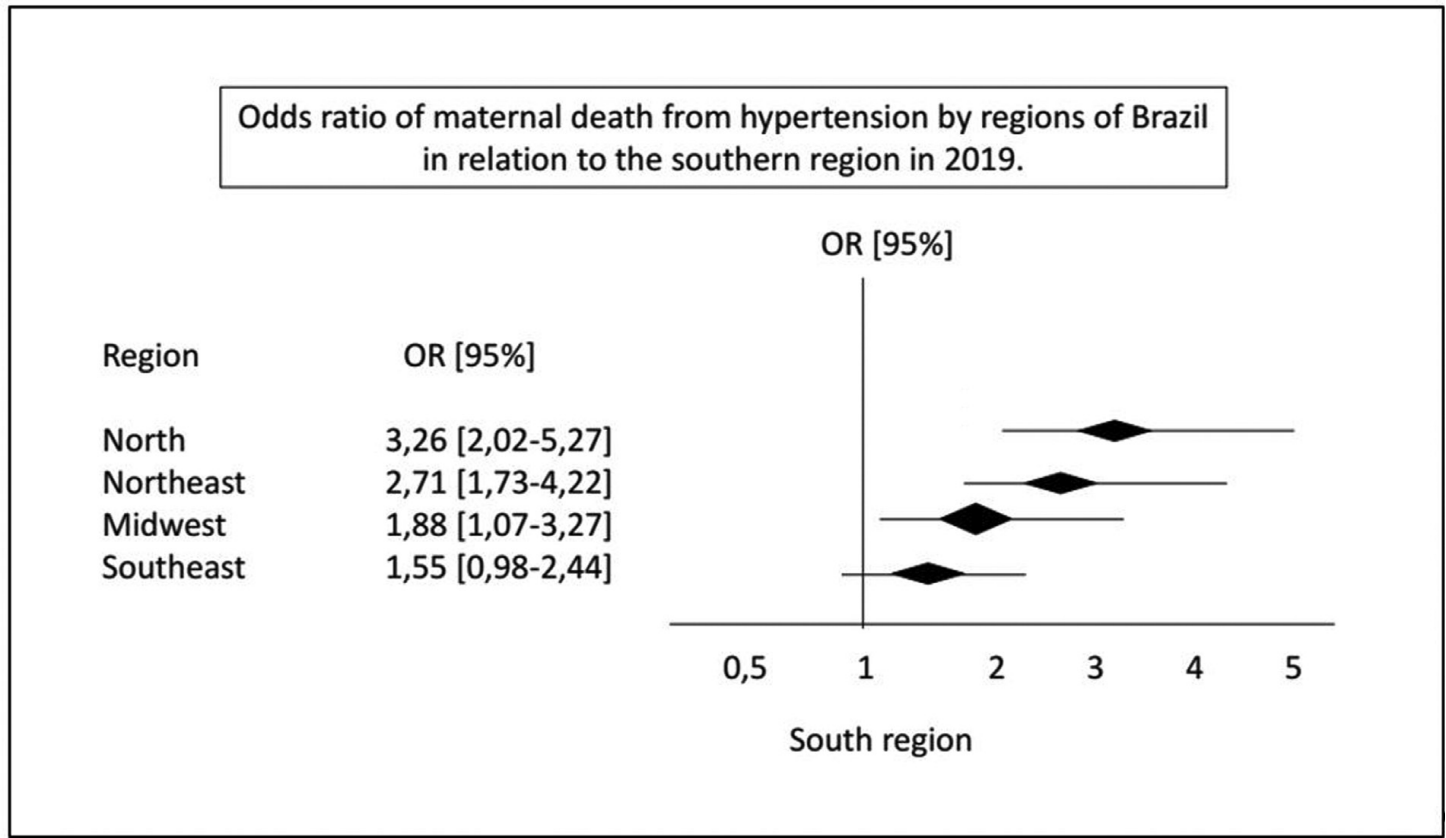
Odds ratio of maternal death from hypertension in pregnancy by regions of Brazil in relation to the southern region in 2019.

When we analyze the ratios of general maternal mortality and death from hypertension and eclampsia in Brazil, we see that we still have a lot to improve. Using data from DATASUS, 3 which are the official data for Brazil and have been properly computed for many years, we found that the maternal mortality ratios due to hypertension have not changed from 2015 to 2019 ( Fig. 1 ). We used the data up to 2019 because they are the last published and revised data that have not yet been contaminated by the mortality of the COVID-19 pandemic. In the comparative analysis of general maternal mortality, from hypertension and from eclampsia, we found an important difference between the Brazilian regions ( Fig. 2 ). The chance of death from eclampsia is 3 times higher in the northern region when compared with the southern region (odds ratio, OR = 3.26; 95% confidence interval, CI: 2.02–5.27) ( Fig. 3 ). The difference in mortality from hypertension during pregnancy among the Brazilians regions is very significant. The eclampsia mortality ratio ranged from 11.47 in the northern region to 2.07 per 100,000 live births in 2019 in the south region ( Fig. 2 ). In the comparative analysis with data from Bartal and Sibai, 1 we found a mortality rate due to eclampsia that managed to be almost zero in some countries.

We believe that the differences in eclampsia incidence and complication rates can be significantly reduced with simple, accessible, and already well-known health measures. Early access to prenatal care, adequate prenatal care, the use of protocols for the management of hypertension and eclampsia, early hospitalization of preeclampsia, indication of pregnancy resolution in cases of preeclampsia with signs of severity or close to term, the prescription of low-dose ASA and calcium supplements for pregnant women at risk for preeclampsia, prophylaxis of eclampsia with magnesium sulfate supplements in the peripartum and antihypertensive treatment are important measures. These measures have a high impact on maternal health and do not require the use of expensive or inaccessible technology. Brazil is immense and with different regional realities in its territories. Locations with greater socioeconomic vulnerability need to be supported and receive customized care for the reduction of their indicators to be achieved.

Although the pathophysiology of preeclampsia has not yet been fully understood and its complete prevention is a difficult task, the occurrence of eclampsia and its consequences can and should be avoided. In the 21st century, we know what to do to treat hypertension during pregnancy and eradicate the deaths caused by it. Doctors gathered in the Brazilian Network for Studies on Hypertension in Pregnancy (RBEHG) have proposed projects with the objective of achieving “zero maternal deaths from hypertension.” The Brazilian Federation of Gynecology and Obstetrics Associations (FEBRASGO), with its continuing education, has been discussing eclampsia in many Congresses and issuing very adequate Protocols, seeking to shed a light on this serious national problem. It is time that risk ranking systems actually work in Brazil and that health care providers for pregnant women (clinicians, family doctors, obstetricians, and nurses) commit to engaging with protocols. Those protocols are already widely known. Despite the difficulties, it encourages us to know that effective and cost-effective actions can be optimized depending on collaboration and an agreement that can guarantee greater safety for all our pregnant women.
